# Biomechanical Analysis of Stoop and Free-Style Squat Lifting and Lowering with a Generic Back-Support Exoskeleton Model

**DOI:** 10.3390/ijerph19159040

**Published:** 2022-07-25

**Authors:** Mark Tröster, Sarah Budde, Christophe Maufroy, Michael Skipper Andersen, John Rasmussen, Urs Schneider, Thomas Bauernhansl

**Affiliations:** 1 Fraunhofer Institute for Manufacturing Engineering and Automation IPA, 70569 Stuttgart, Germany; sarahbudde@googlemail.com (S.B.); christophe.maufroy@ipa.fraunhofer.de (C.M.); urs.schneider@ipa.fraunhofer.de (U.S.); thomas.bauernhansl@ipa.fraunhofer.de (T.B.); 2 Institute of Industrial Manufacturing and Management IFF, University of Stuttgart, 70569 Stuttgart, Germany; 3 Department of Materials and Production, Aalborg University, 9220 Aalborg, Denmark; msa@mp.aau.dk (M.S.A.); jr@mp.aau.dk (J.R.)

**Keywords:** biomechanics, musculoskeletal modeling, occupational exoskeletons, ergonomics, manual handling

## Abstract

Musculoskeletal disorders (MSDs) induced by industrial manual handling tasks are a major issue for workers and companies. As flexible ergonomic solutions, occupational exoskeletons can decrease critically high body stress in situations of awkward postures and motions. Biomechanical models with detailed anthropometrics and motions help us to acquire a comprehension of person- and application-specifics by considering the intended and unintended effects, which is crucial for effective implementation. In the present model-based analysis, a generic back-support exoskeleton model was introduced and applied to the motion data of one male subject performing symmetric and asymmetric dynamic manual handling tasks. Different support modes were implemented with this model, including support profiles typical of passive and active systems and an unconstrained optimal support mode used for reference to compare and quantify their biomechanical effects. The conducted simulations indicate that there is a high potential to decrease the peak compression forces in L4/L5 during the investigated heavy loaded tasks for all motion sequences and exoskeleton support modes (mean reduction of 13.3% without the optimal support mode). In particular, asymmetric motions (mean reduction of 14.7%) can be relieved more than symmetric ones (mean reduction of 11.9%) by the exoskeleton support modes without the optimal assistance. The analysis of metabolic energy consumption indicates a high dependency on lifting techniques for the effectiveness of the exoskeleton support. While the exoskeleton support substantially reduces the metabolic cost for the free-squat motions, a slightly higher energy consumption was found for the symmetric stoop motion technique with the active and optimal support mode.

## 1. Introduction

A total of 58% of all workers in the European Union reported musculoskeletal disorders (MSD) in 2015 [1]. Among those, pain in the back (46%) and muscles of the shoulders, neck, and upper limbs (43%) were the most commonly reported [1]. Significant physical risk factors include working in awkward postures, moving and carrying heavy objects, and repetitive motions [1]. In order to prevent MSDs, working conditions should be improved first by the ergonomic design of working environments and by training or instruction measures in a second step [2].

**Figure 1:**
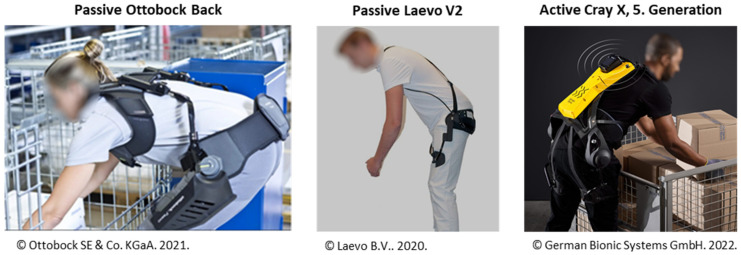
Selection of commercial back-support exoskeletons (BSEs) applying supportive torques aligned with the user’s hips [12,14,17].

However, some lines of work (e.g., in agriculture, construction, and logistics) cannot be linked to defined locations; environmental requirements can vary, or sufficient technical or organizational measures are not applicable for other reasons [3]. In those cases, occupational exoskeletons can be an effective and flexible ergonomic improvement [3]. These wearable mechanical systems are meant to reduce the physical stress of workers, utilizing passive or active forces and/or torques generated by elements such as springs or active actuators [4]. In the last few decades, a variety of potential exoskeleton solutions for different industries were proposed, supporting either the lower, upper or the whole body [5,6,7,8,9,10,11]. These include some passive and a few active systems to support the lower back while bending forward and lifting heavy objects (e.g., Paexo Back [12], Model Y [13], Cray X [14], Muscle Suit [15], Flex Lift [16], Laevo V2 [17], exoBack [18], and BackX [19]). Two passive and one active variant of them are shown in Figure 1.

Recently, many industries have started to integrate exoskeletons into logistic procedures and production lines to reduce physical load and to improve workers’ safety [20]. Holistic ergonomic assessment methods have been introduced to support their implementation in the work field [21,22]. Hence, it seems obvious that a better insight into the biomechanical effects resulting from the use of exoskeletons is needed. For that purpose, approaches based on biomechanically accurate multi-body simulations can be used to analyze and optimize the effects of exoskeletons on the biomechanics of the individual human body during different tasks and loadings [23,24,25]. For instance, Tröster et al. recently proposed a musculoskeletal model-based exoskeleton design framework in which the motion data of manual tasks are considered to assess body loads utilizing refined multi-body simulations [26]. Analyses of the resulting body loads help design and assess exoskeletons that can relieve highly loaded body areas [26]. Subsequent simulations of the joint exoskeleton and human models were conducted in order to optimize the designs and validate their biomechanical effects on the body [25].

To optimally implement occupational exoskeletons, a general ergonomic agreement is that high peak loads should be reduced as much as possible to decrease the risk of musculoskeletal injuries. However, in addition, high cumulative loads, caused by long load durations or many repetitions of load-bearing activities, can also lead to lasting musculoskeletal health issues for affected workers [27]. Both peak and cumulative loads are affected by back support exoskeletons (BSEs) and should be investigated to understand hazards and prevent mechanical muscle, bone, tendon, and cartilage damage. Furthermore, to successfully implement BSEs, it is highly important to understand how they biomechanically can assist, e.g., as existing systems have different support intensities within various BSEs [28,29,30], whilst considering the effect of lifting techniques [31], handling weights, and application-given boundary conditions. However, there are further working conditions related to MSDs for which existing commercial solutions might not be applicable. These conditions should be understood, considering their biomechanical effectiveness. In addition, while exoskeletons have the potential to reduce muscular demands [4,32], their ability to prevent MSDs is not yet conclusive [33]. The redistribution of load from one body part to another can potentially introduce new health risks [3]. Furthermore, Van der Have et al. [34] found in their study with ten participants lifting two different weights with two lifting techniques that squat lifting imposes higher peak full body musculoskeletal loading and similar low back loading compared to stoop lifting, reflected in peak moments; peak joint power in L5/S1; and peak EMG signals for nine hip, back, and shoulder muscles. This should be further understood in combination with varying exoskeleton support. Furthermore, it is important to understand the effect of BSEs with hip-aligned torques on the human metabolism [35], as they are more effective during lifting than knee or ankle torque assistance [36]. Kim et al. analyzed and stated, based on a combined exoskeleton–human model, the optimal level of assistance in combination with the required weight for a BSE in a symmetric, free-squat lifting motion [37]. Nevertheless, the metabolic effect of hip assistance on other lifting techniques is not yet clearly understood.

Therefore, the aim of the present model-based analysis was to demonstrate a generic approach for investigating the influence of BSE support characteristics on the biomechanical intended main effects of relieving lower back loading and metabolic cost for an example subset of manual handling motions and loading conditions based on a sophisticated musculoskeletal model. For that purpose, sixty motion cycles were captured and used to analyze dynamic load lifting and lowering, executed with two different lifting techniques, performed both symmetrically and asymmetrically. The generic exoskeleton model was designed based on existing BSEs with hip-aligned support torques (Figure 1). Furthermore, two passive and active support modes were modeled and compared with a biomechanically idealized support mode as an optimal assistance reference.

## 2. Methods

### 2.1. Experimental Setup

The data for the biomechanical models were acquired in the motion laboratory at Fraunhofer IPA in Stuttgart, Germany. The male test subject had an age of 28 years, a bodyweight of 80.9 kg, and a height of 1.82 m. At the time of measurement, the subject was healthy and had no orthopedic restrictions in the lower back area. The subject did not engage in any intense physical activity immediately before the motion capture and was used to heavy manual lifting in a sportive context. After an explanation of the test procedure, the subject signed a declaration of consent. After a training session, the subject executed a walking sequence and all tasks with five repetitions.

**Figure 2:**
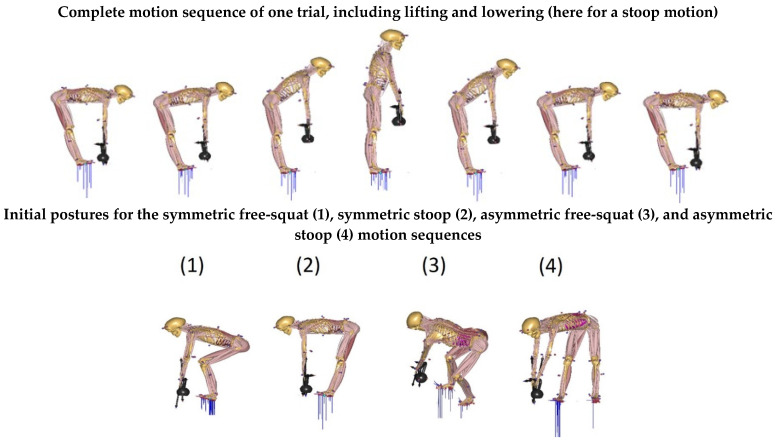
Illustrations of measured symmetric and asymmetric motion sequences.

The dynamic trials were designed as an entire motion cycle, which consisted of picking up a kettlebell, returning to an upright position while holding the kettlebell, and then lowering the kettlebell back to the ground. The subject was instructed to lift and lower in a normal, controlled manner and to perform all repetitions at a similar speed. Throughout the entire motion cycle, the arms were extended straight downwards. Furthermore, the subject was instructed to execute all dynamic motions in stoop and free-squat motion techniques. The subject was also instructed to keep a straight trunk, and, for the free-squat motion, the subject was allowed to move his legs in whatever way felt most comfortable. For the stoop motions, the subject was asked to flex his knees as little as possible. For all trials, three kettlebells of different weights (4 kg, 8 kg, and 20 kg) were used. The kettlebells were centrally placed 10 cm in front of the subject’s feet for symmetrical motions; for asymmetric motions, the kettlebell’s initial position was 10 cm in front and 50 cm to the right-dominant side of the subject (Figure 2).

### 2.2. Collection and Processing of the Motion Data

The motion data were collected using a motion capture system from Qualisys (Qualisys AB, Gothenburg, Sweden), with 15 Oqus cameras at a frame rate of 100 Hz. A total of 38 retro-reflective markers were placed on the subject’s entire body, mainly on bony landmarks, based on the marker set-up of the Anybody Modeling System (AMS). The motion of the kettlebells was recorded using a set of four markers for each kettlebell. The trajectories of all markers were processed with Qualisys (QTM, Qualisys AB, Gothenburg, Sweden; version 2020.3), using a gap-filling method of cubic polynomial interpolation when required. The trajectories were further improved by smoothing them using a Butterworth filter with a cut-off frequency of 10 Hz.

One of the kettlebell markers was used to determine the starting and ending reference for the lifting and lowering movements in order to ensure comparability between all motion sequences. All sequenced trials were exported in C3D format (C3D.org, 2021) to be further processed in AMS (version 7.3.1).

### 2.3. Human Musculoskeletal Model and Model of the External Load

The human musculoskeletal model in the AMS is based on the full-body AnyMocap model taken from the AnyBody Managed Modeling Repository (AMMR, version 2.2.0), including the Spine Model consisting of a pelvis segment, five lumbar segments, and one thorax segment, as the rib cage and all spine segments are connected by six spherical joints. The spine model was mainly developed by De Zee et al. [38], and validated by Bassani et al. [39]. The parameter identification, kinematics, and inverse dynamics of all the trials were solved within the AMS [40]. The anthropometrical length parameters of the human model were identified based on the weight and height of the subject and a captured walking sequence with an average speed with the combination of motion capture-based model parameter identification [41] and the so-called length–mass–fat scaling method [42]. Kinematics were solved by applying the optimization methods of Andersen et al. [43]. The ground reaction forces were predicted based on the method of Fluit et al. [44], which was validated by Larsen et al. [45] for a variety of manual handling movements.

The inverse dynamics were solved within the muscle recruitment of the AMS by finding the optimal muscle activations for a given dynamic scenario. The muscle recruitment solves an optimization problem with an objective function *G*, which tries to minimize the normalized forces $f^{\left(M\right)},f^{\left(C\right)},f^{\left(R\right)}$ (1) while honoring the dynamic equilibrium Equation (2) and keeping non-negative constraints, e.g., that the muscles can only pull (3).

(1) $$min\;G\left(f^{\left(M\right)},f^{\left(C\right)},f^{\left(R\right)}\right)=\sum_{i=1}^{n^{\left(M\right)}}{\left(\frac{f_{i}^{\left(M\right)}}{N_{i}^{\left(M\right)}}\right)}^{3}+\sum_{i=1}^{n^{\left(C\right)}}{\left(\frac{f_{i}^{\left(C\right)}}{N_{i}^{\left(C\right)}}\right)}^{3}+\sum_{i=1}^{n^{\left(C\right)}}{\left(\frac{f_{i}^{\left(R\right)}}{N_{i}^{\left(R\right)}}\right)}^{3}$$

(2) Cf=d,

(3) $$0\leq f_{i}^{\left(M\right)},i=1,\ldots ,n^{\left(M\right)};0\leq f_{i}^{\left(C\right)},i=1,\ldots ,n^{\left(C\right)};0\leq f_{i}^{\left(R\right)},i=1,\ldots ,n^{\left(R\right)}$$

The objective function *G*, which tries to minimize the muscle $f_{i}^{\left(M\right)}$, contact $f_{i}^{\left(C\right)}$, and residual forces $f_{i}^{\left(R\right)}$ with strengths $N_{i}^{\left(M\right)},N_{i}^{\left(C\right)},N_{i}^{\left(R\right)}$, is implemented in the form of the polynomial criterion of third order [40]. The strengths of the contact forces are defined as very high ($N_{i}^{\left(C\right)}=10,000$) compared to the muscle forces to prioritize mechanical contact much more than muscle activation. The residual forces are less strong ($N_{i}^{\left(R\right)}=2\ldots 5$) than the contact elements and are grounding the pelvis segment to improve numerical stability. The ground reaction forces are considered as contact forces $f_{i}^{\left(C\right)}$ within the muscle recruitment [45]. The dynamic equilibrium equations are described with *C* as the coefficient matrix, *f* as all of the unknown muscle and joint reaction forces, and *d* as all of the external and inertial forces.

**Figure 3:**
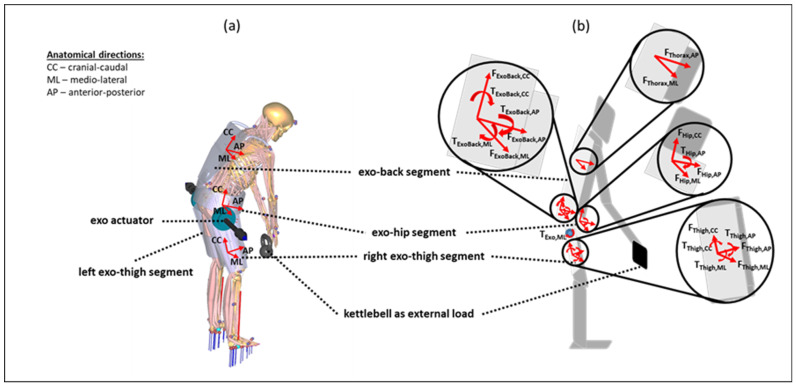
Generic exoskeleton–human model with anatomical directions as the basis for the orientations of the axes of rotation and translation for the interaction forces and moments. The model is illustrated with anatomical directions (**a**) and implemented interaction forces and moments (**b**) based on Table 1. Further, the axes of the actuator torques are outlined.

The three kettlebells were modeled as three-dimensional objects with realistic geometries (Figure 3) and uniform mass distributions with SolidWorks (Dassault Systèmes, Vélizy-Villacoublay, France; version Premium 2019 SP 4.0) and added via a software interface (SolidWorks2AnyBodyExporter) in the AMS. The kinematics of the kettlebells were solved based on the recorded marker positions. The interaction kinetics were solved by adding reaction forces and moments in all directions between both hand and kettlebell segments, which are considered as further contact forces $f_{i}^{\left(C\right)}$ within the muscle recruitment.

### 2.4. Generic BSE Model

The generic BSE model consists of four rigid segments that are kinematically constrained each to a human thigh, hip, or thorax segment. Therefore, each of the four exoskeleton segments is kinematically fully constrained to the corresponding human segment (exo-thigh segments to femur bones, exo-hip segment to pelvis bone, and exo-back segment to rib cage). Interaction forces and moments are defined separately in plausible directions between each exoskeleton and human segment (Table 1).

**Figure 4:**
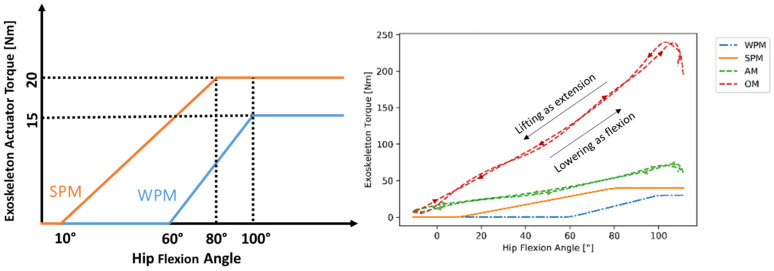
Characteristic torque–angle curves for WPM, SPM (**left** graph), and all modes, including summed torque for both actuators for a symmetric, free-squat motion with 20 kg over flexion and extension phase (**right** graph).

The exoskeleton support is modeled using two torque elements each aligned to the ipsilateral human hip joint in a medial–lateral direction, perpendicular to the sagittal plane, and acting between the exo-thigh and exo-hip segments (Figure 3). The actuator torque elements are described in Table 1 as two interaction moments (exo-hip segment <> exo-thigh segment). Three support modes with different torque characteristics were defined and considered in the analysis to mimic the characteristics of the main categories of the existing BSEs as hip-support exoskeletons: a weak and a strong passive mode (WPM, SPM, respectively), and an active support mode (AM) [28,29,30]. Furthermore, an idealized fourth support mode, coined the optimal support mode (OM), was considered as the baseline. These four modes are represented in Figure 4.

For the passive support modes, the torque was initially zero before reaching a certain hip flexion angle threshold; then, it increased linearly at an angle, until the hip angle reaches a second threshold, after which the torque remains constant. The angle thresholds and the progression rate of weak (WPM) and strong (SPM) passive modes were chosen to approximate the characteristics of the two example variants of existing systems, which can vary in the intensity of applied torque and bending angle offset, to understand potential biomechanical differences between passive variants [29,30].

On the other hand, the active and optimal characteristics are defined as torque elements $f_{i}^{\left(AM,OM\right)}$ with limited ($N_{i}^{\left(AM\right)}=40\;\mathrm{Nm}$) and unlimited strength ($N_{i}^{\left(OM\right)}=\infty \;\mathrm{Nm}$), which was respected in the muscle recruitment. The OM defines a probably unrealistic and theoretical optimal support reference for the exoskeleton torque actuators embedded in the described interaction kinetics architecture (Figure 3, Table 1) for biomechanical support allocation. The AM, instead, mimics an optimally controlled active support by supplying exactly the right amount of torque at the right time. Thus, the AM’s and OM’s torque quantities were computed independently from the hip joint angles. All actuator torques were considered as dynamic torque elements within the muscle recruitment in the AMS.

The weight of each exoskeleton model is defined based on existing, proposed, and theoretical assumptions for support solutions (WPM: 1 kg; SPM: 3 kg; AM: 6 kg; and OM: 0 kg). The weight of each BSE mode was modeled based on realistic weight estimations for each exoskeleton segment, including inertia properties based on the modeled CAD geometries among the exoskeleton segments (Figure 3).

### 2.5. Biomechanical and Ergonomic Parameter Investigation

Based on the aforementioned optimization approach in the AMS, all muscle and joint reactions were computed. Furthermore, metabolic energy consumptions (costs) were approximated based on the quantified muscle forces in the AMS. The metabolic costs were computed as the integral over time of the metabolic power *P_met_* (4), based on the mechanical power *P_m_* of each modeled muscle and dependent on a differing factor for eccentric $e_{ecc}$ and concentric $e_{con}$ muscle contraction (5) [46].

(4) $$P_{met}=\frac{P_{m}}{e},\;\mathrm{where}\;e=\left\{ \begin{array}{c} e_{con}\;if\;v^{ce}\leq 0\\ e_{ecc}\;if\;v^{ce}>0 \end{array}.\right.$$

(5) $$e_{con}=0.25\;\mathrm{and}\;e_{ecc}=-1.25$$

As the preliminary biomechanical characterization of the lumbar loading, the lumbar joint moments of the L4/L5 joint were outlined for all four motion techniques and the maximal weight of 20 kg. Furthermore, as presented in the Section 3, the load sequences were investigated without exoskeleton support to understand the biomechanical effects of ergonomic parameters such as external weight, symmetry, and motion technique. As indicators of injury risk in the lower back, peak and cumulative L4/L5 compression and shear, indicated in peak force and impulse, were analyzed. Furthermore, metabolic energy consumptions for the whole body were considered to gain more insight into the mechanical body–work effort. The results of the analysis with the exoskeleton are placed in the Section 3.2, in which L4/L5 peak compression and modeled metabolic consumptions were considered. All biomechanical parameters were quantified as arithmetic means with standard deviations for the five trials for each motion sequence and exoskeleton support mode. In the Supplementary Materials, the lumbar extension joint moments for all motions and the axial rotation moments for the asymmetric motions are, as an example, outlined for the external weight of 20 kg without support in comparison with all exoskeleton support modes.

## 3. Results

### 3.1. Biomechanical Analysis without Exoskeleton

**Figure 5:**
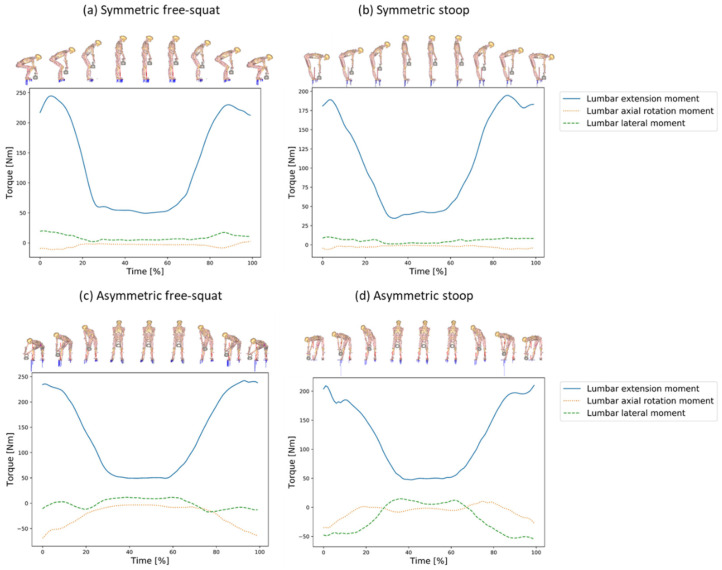
Lumbar joint moments for all motion techniques and external weight of 20 kg illustrated together with the human model in sagittal (**a**,**b**) and frontal (**c**,**d**) plane as mean moments for all five trials over normalized time.

For the symmetric motions, the lumbar axial rotation and lateral moments were relatively small Figure 5a,b compared to the asymmetric ones Figure 5c,d. The free-squat motion technique raises slightly higher extension moments Figure 5a than the stoop technique Figure 5b,d. High lumbar axial rotation moments occurred for the asymmetric motion techniques Figure 5c,d. Especially for the asymmetric stoop, substantial high lumbar lateral moments appeared Figure 5d.

**Figure 6:**
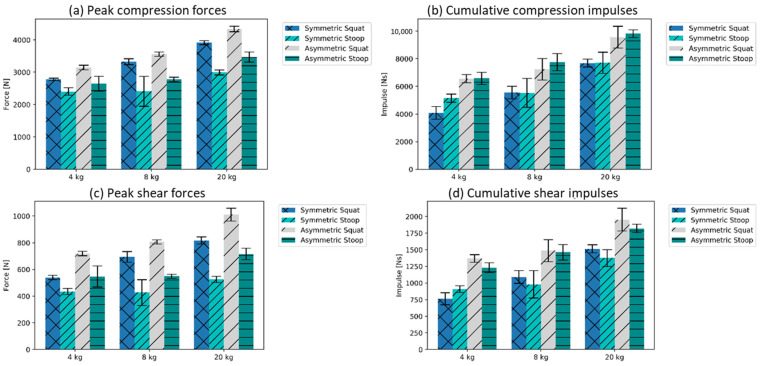
Forces and impulses in L4/L5 joint (± standard deviation (SD)).

The L4/L5 peak compression forces were found to be generally higher for the free-squat motion technique than for the executed stoop motion technique (Figure 6a, mean of sym., and asym. squat 3509 ± 68 N; mean of sym. and asym. stoop: 2783 ± 184 N). The cumulative spinal loads, quantified using the L4/L5 compression impulse, were higher for the asymmetric motions than for the symmetric motions with the same loading conditions (Figure 6b, symmetric motions’ mean: 5955 Ns ± 553 SD; and asymmetric motions’ mean: 7925 Ns ± 534 SD). Similar trends were identified for the shear forces (Figure 6c: L4/L5 shear peak force, mean of sym. and asym. squat: 765 N ± 27 SD; mean of sym. and asym. stoop: 533 N ± 47 SD; Figure 6d: L4/L5 compression impulse, symmetric motions’ mean: 1106 Ns ± 106 SD; and asymmetric motions’ mean: 1554 N ± 108 SD).

**Figure 7:**
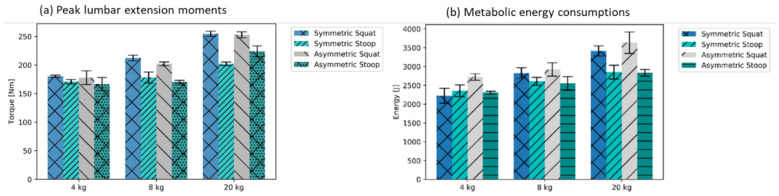
Mean peaks over five trials for lumbar extension moments and metabolic costs, both approximated based on the modeling assumptions in the AMS (±SD).

Higher maximal extension moments occurred for all free-squat motion techniques (mean maximum of symmetric and asymmetric free-squat: 213 Nm ± 5 SD). In contrast, for stoop motion techniques, the moments were substantially lower (mean maximum of symmetric and asymmetric stoop: 185 Nm ± 7 SD) (Figure 7a). Furthermore, the squat motions induced higher mean metabolic energy consumption than motions performed with the stoop technique (Figure 7b, mean of sym., and asym. squat: 2958 N ± 172 SD; mean of sym. and asym. stoop: 2584 N ± 127 SD). The relative difference between the stop and squat slightly increased with the amount of external load. For the handling of the 4 kg kettlebell, a slightly higher mean value for the stoop motion technique in symmetric motions in the metabolic energy consumptions were recorded (Figure 7b).

### 3.2. Analysis with Generic Exoskeleton Support

**Figure 8:**
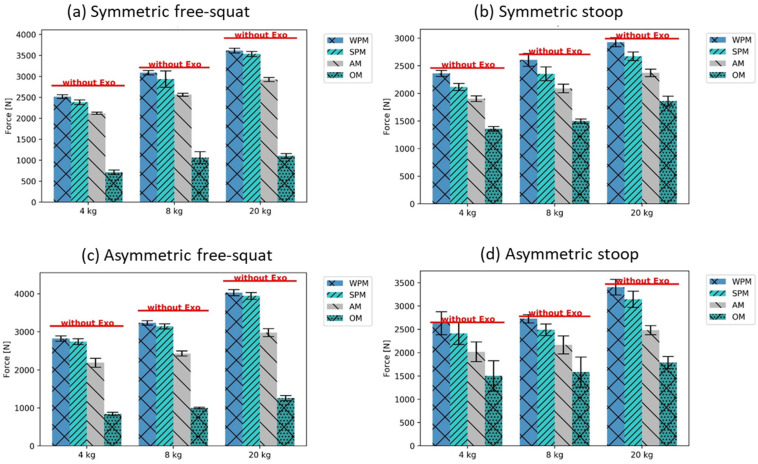
L4/L5 peak compression forces with the different exoskeleton support modes (±SD), compared to the reference as mean without exoskeleton support.

Considering first the load on L4/L5, the OM support mode reduced the peak compression forces the most, followed by AM, SPM, and WPM (Figure 8). This tendency was observed for all motions and loads. Comparing the results for the free-squat (Figure 8a,c) and stoop (Figure 8b,d), when using OM, the peak compression force was lower for the free-squat than for the stoop (mean of sym. and asym. squat: 998 N ± 64 SD; mean of sym. and asym. stoop: 1599 N ± 158 SD), although the opposite was found without exoskeleton support. In contrast, for the three other modes, the peak compression forces remained higher during the stoop than during the free-squat, for both the symmetric and asymmetric motions. The difference in peak compressive force reduction between the WPM and SPM was higher for the stoop than for the free-squat motion technique, independent of the symmetry of motions (mean of L4/L5 peak compression force difference between WPM and SPM, free-squat motions: 102 N ± 31 SD; stoop motions: 244 N ± 7 SD). For the free-squat motions, a mean reduction in L4/L5 peak load of about 9.8% for all weights was found with the modelled passive support modes (peak compression force with averaged WPM and SPM: 3165 ± 75 N); AM reduced mean loads by 27.7% and OM reduced mean loads by 71.6%, compared to those recorded without the exoskeleton (mean of peak compression forces for the free-squat motions, AM: 2536 ± 66 N; OM: 998 ± 64 N; without exo: 3339 ± 63 N). In comparison, for the stoop motions, a mean reduction in L4/L5 peak loading of only 4.7% for all weights was found with the modelled passive support modes (mean of peak compression forces of WPM and SPM: 2655 ± 130 N), with 22.0% for AM and 42.6% for OM, compared to those recorded without the exoskeleton (mean of peak compression forces for the stoop motions, AM: 2172 ± 116 N; OM: 1599 ± 158 N; without exoskeleton: 2784 ± 185 N).

**Figure 9:**
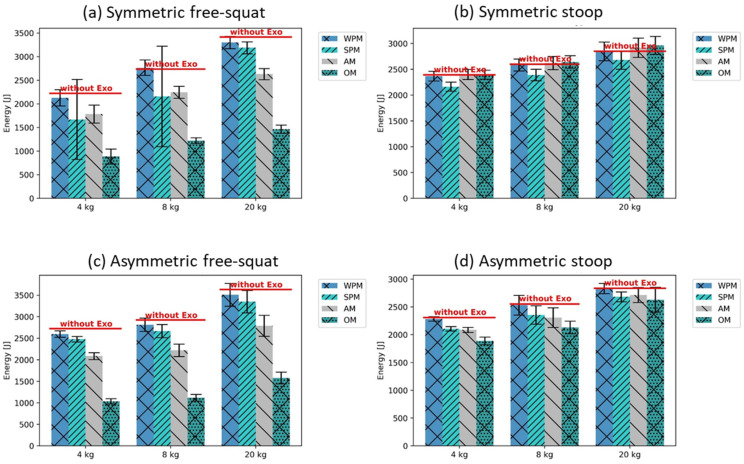
Metabolic costs with the different exoskeleton support modes (±SD), compared to the reference as mean without exoskeleton support.

For the free-squat, similar tendencies regarding the influence of the support modes on metabolic energy consumption, as found previously for compression forces, i.e., the strongest reduction with OM, followed by AM, SPM, and WPM, were recorded (mean difference of metabolic energy reduction between WPM and OM, squat motions: 1634 ± 63 J; stoop motions: 133 ± 15 J) (Figure 9). A notable exception was the symmetric free-squat with the 4 and 8 kg weights, in which the SPM supported more than the AM, but this had a high standard deviation (Figure 9).

In contrast, for the stoop, the energy consumption with the AM and OM was only slightly lower (for asymmetric motions), or even higher (for symmetric motions) than with SPM (metabolic energy for symmetric stoop motions, mean of WPM and SPM: 2505 ± 128 J; mean of AM and OM: 2657 ± 132 J) (Figure 9).

## 4. Discussion

The purpose of the demonstrated generic and abstract analysis approach is to enable systematic biomechanical comprehension of the effectiveness of BSEs with hip-aligned torque embedded in an understanding without the use of an exoskeleton. The exoskeleton support assessment reveals effects for different motion techniques and external loads in a lifting and lowering motion sequence. The effects are mainly expressed in the L4/L5 back loading in compression and shearing. Biomechanical loadings are considered in peak and cumulative occurrences. In addition, the model-based metabolic energy consumptions for all motions with and without exoskeleton are considered.

As expected, in the present analysis, none of the compression peak forces exceeds the recommended age- and gender-specific Dortmund Revised Reference Values of Jaeger [47] (5400 N) for a 28-year-old male. Nevertheless, it should be kept in mind that women over 40 years old and men over 50 years old (compression force limit: 3100 N) should not execute any of the performed free-squat motions with 8 and 20 kg, stoop motions with 20 kg, and asymmetric free-squat motions with 4 kg. Furthermore, the results without the exoskeleton indicate that the free-squat motions conducted in this study induce higher compression forces on L4/L5 than the stoop motion technique. The lumbar joint moments confirm the characteristic difference between both motion techniques (Figure 5). In addition, the quantified shear peak loads confirm the different loading between the symmetric and asymmetric motions. The instructed free-squat motions induced a longer lever arm regarding the horizontal distance between the pelvis and kettlebell than during the stoop motions. This is the case as the free-squats were performed adversely based on geometrical firmly defined conditions to ensure a comparable “kettlebell to feet” distance for both motion techniques, as it can appear in industrial work environments. Under ideal ergonomic conditions, the lever arm would be much shorter as the kettlebell would be placed exactly between both feet on the ground at the beginning and ending of the task. These firmly defined boundary conditions should be taken into account and hinder a general conclusion for the comparison of different motion techniques. However, this trend decreases considering the compression impulses for the asymmetric motions, which can be explained by an increased time duration of asymmetric compared to symmetric motion sequences. The cumulative load is higher, as the impacting force’s duration is longer for asymmetric motions, compensating for differences between the stoop and free-squat motion techniques when performed asymmetrically. The quantified metabolic costs confirm the findings of Wang et al. [48] that the squat motions—in our case, free-squat motions—induce a higher energy consumption than stoop motions, which is not surprising, because the squat motion lifts almost the entire body weight in addition to the kettlebell, while the stoop includes only a fraction of the body weight.

Considering the exoskeleton support modes, they indicate, as expected, that OM reduces the lower back peak loading the most, followed by AM, SPM, and WPM for all motions and external weights. The results indicate that hip-aligned back-support exoskeletons reduce L4/L5 peak loading stronger for the free-squats conducted than for the stoop motions. Furthermore, it should be noted that the WPM and SPM, representative of the support provided by passive exoskeleton systems, support differently during free-squat motions than in the stoop motion technique. The WPM provides stronger support for free-squat motions, as the difference between the WPM and SPM is considerably less than for stoop motions where the SPM has a stronger relieve effect (Figure 8), which can be explained by the modelled hip flexion–torque dependency. In using similar biomechanical modeling techniques, Schmalz et al. [49] identified reductions of 21% for the L4/L5 peak compression force for a passive exoskeleton, which is very dependent on the modeled torque characteristic, motion technique, and handled load (in their case, a box of 10 kg). Furthermore, the statement by van der Have et al. [34] that precisely executed squat technique can induce high peak loads to the lower back is not amplified by the exoskeleton support modes, as found for the free-squat motions performed in the present analysis. Here, the exoskeleton support protects the vulnerable lower back by reducing the loading (Figure 8). 

Considering the metabolic energy consumption, an interesting finding of the present analysis is that the applied exoskeleton support reduces the metabolic costs of free-squat motions more than for stoop motions (Figure 9). The metabolic energy saved thanks to the exoskeleton support should be for the lower back muscles, almost similar for both motion techniques for all weights and exoskeleton support modes. The difference observed could be related to the change in metabolic energy, as this is consumed by the muscles of the lower extremities. The findings of the analyzed metabolic costs forecast a natural alignment of the motion technique from the ergonomically unfavorable stoop to a more squat-like motion technique provoked by BSEs with hip-aligned torque. The tendency found supports the assumption that, with higher knee flexion of the worker, more metabolic energy can be saved by the exoskeleton support. This finding could overcome the struggle of ergonomists persuading workers to perform lifting and lowering using the squat technique, instead of the stoop technique, since the human body tends to favor energy-efficient motions in the long term.

As a major limitation of this generic approach, by applying an idealized BSE model, the effects of misalignment, hysteresis, and parasitic forces are simplified. It should be kept in mind that connecting the exoskeleton with the human body can be a tough task. Misalignment between the human and the artificial joints can cause unplanned and undesired forces, adversely affecting user comfort. Therefore, a system with the least negative side effects is desirable. One approach to minimize the misalignment between the human and the artificial joints is to initially align the exoskeleton joints with the corresponding anatomical joint, either manually or by mechanisms. The challenge of this concept is that, although it may be aligned at first, any shifting of the exoskeleton caused by the movement of the human body can lead to undesired interaction forces. It is important to minimize these relative movements between the exoskeleton and the user, while small misalignments can be taken into account. To pursue this approach, additional kinematic structures, for example, joints, sliders, or elastic elements, are added to the existing exoskeleton solutions [28,30]. Hence, detailed hysteresis modeling of the passive exoskeleton support is neglected in the present analysis approach, which has a notable biomechanical effect in existing passive solutions [28,30,50]. As a further limitation of the present analysis, only one subject’s motions were investigated, and no individual spine movements were captured, which would be possible with more motion markers for an individual understanding of the spine loading [51]. In addition, the support modes of the exoskeleton were modeled on the motions detected without an exoskeleton, which means the kinematic adjustments that an exoskeleton can cause are neglected [52]. The assumption that the muscle recruitment strategy for all exoskeleton support modes remains the same and recruits for AM the ideal support torques, which is currently not possible with existing active torque controllers, is a further limitation, which should be investigated. Considering the metabolic costs, it should be kept in mind that they are quantified based on simplistic metabolic modeling that needs to be refined in future studies based on existing research in this field [53,54]. Finally, all results are investigated without considering statistical significance, as the demonstrated generic modeling approach will be used in future studies with more trials and subjects with expected statistical relevant outcomes.

Even so, the observed biomechanical trends can inform exoskeleton developments, implementations, and assessments. Nevertheless, further validation and verification of the generic analysis approach by more motions, subjects, and in-detail implemented exoskeleton mechanisms are needed for practical estimation.

## 5. Conclusions

A generic analysis approach was developed and demonstrated to gain a combined understanding of ergonomic manual handling parameters such as motion technique, motion symmetry, external load, and how they biomechanically interact with various exoskeleton support modes. More specifically, the generic approach can enable the investigations of the biomechanical effects of BSEs with hip-aligned torque for different external loads and motion techniques.

The analysis quantified L4/L5 back loading, which was further used for comparison with different exoskeleton support modes. The results without the exoskeleton for the conducted free-squat motions induced higher back loading than for the stoop motions, supporting the necessity for in-detail biomechanical model assessments with application-specific motion data. The four modeled exoskeleton support modes reduced L4/L5 back loading in the expected order of WPM, SPM, AM, and, most strongly, OM. Furthermore, all of the models, and especially the modeled optimal and active assistances (OM and AM), indicated that for squat-like motions, the applied support characteristic has a higher relief impact on the lower back than for stoop motions. The theoretical and optimal assistance reference (OM) reduced the L4/L5 back loading substantially more for the free-squat than for the stoop motions. The analyzed metabolic energy consumptions confirmed this trend, which supports the assumption that hip-aligned BSEs can optimize the motion technique of workers during manual lifting and lowering.

In general, the modeling approach seems suitable for the systematic analysis of exoskeleton variants in the modern human-centric workplace. Inevitably, this approach needs to be further evaluated with more subjects and application-related motion data to determine the practical estimation, to answer the question of how anthropometrics and application flexibility can play a role in the efficacy of industrial BSEs.

## Figures and Tables

**Table 1:** Overview of all bidirectional interaction forces and moments between the exoskeleton and human segments in the rigid multi-body system. In total, 14 translational interaction forces and 12 interaction moments are modeled. The directions of the forces and moments are defined on the basis of the anatomical orientation of the axes of rotation (Figure 3).

Kinetic Interfaces	Interaction Forces (14)	Interaction Moments (12)
human femur <> exo-thigh segment (2 × (left and right))	F_Thigh,CC_ F_Thigh,ML_ F_Thigh,AP_	T_Thigh,CC_ T_Thigh,ML_ T_Thigh,AP_
human pelvis <> exo-hip segment	F_Hip,CC_ F_Hip,ML_ F_Hip,AP_	T_Hip,AP_
human thorax <> exo-back segment	F_Thorax,CC_ F_Thorax,AP_	-
exo-hip segment <> exo-back segment	F_ExoBack,CC_ F_ExoBack,ML_ F_ExoBack,AP_	T_ExoBack,CC_ T_ExoBack,ML_ T_ExoBack,AP_
exo-hip segment <> exo-thigh segment (2 × (left and right))	-	T_ExoActuator,ML_

## Data Availability

The data presented in this analysis are available on request from the corresponding author.

## Supplementary Materials

The following supporting information can be downloaded at: https://www.mdpi.com/article/10.3390/ijerph19159040/s1, Figure S1: Lumbar extension moments for all motions (a–d) and axial rotation moments for asymmetric motions (e,f) with external weight of 20 kg without support and for all support modes (meaned for all five trials over normalized time).
