# Supplementary material for: Biomechanical Analysis of Stoop and Free-Style Squat Lifting and Lowering with a Generic Back-Support Exoskeleton Model
Source: Int J Environ Res Public Health. 2022 Jul 25;19(15):9040. doi: 10.3390/ijerph19159040 (PMC9332239; doi:10.3390/ijerph19159040)
Supplement: Supplementary file 1 [file ijerph-19-09040-s001.zip › ijerph-1721756-supplementary.pdf]

International Journal of
Environmental Research and Public Health

MDPI

Supplementary Material

Biomechanical Analysis of Stoop and Free-Style Squat Lifting and Lowering with a Generic Back-Support Exoskeleton Model

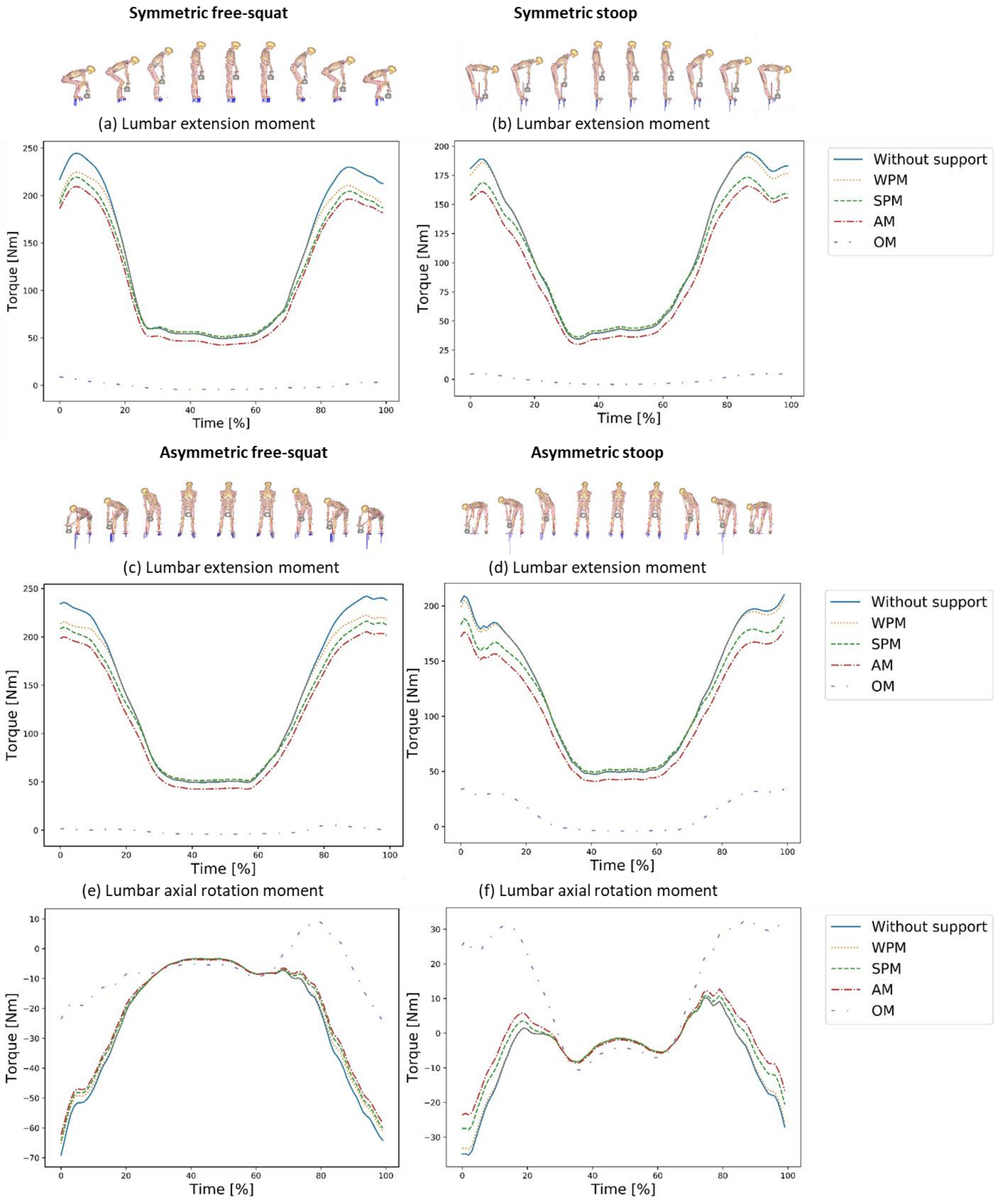

Figure S1. Lumbar extension moments for all motions (a,b,c,d) and axial rotation moments for asymmetric motions (e,f) with external weight of 20kg without support and for all support modes (meaned for all five trials over normalized time).
